# Radically resected node-negative squamous cell carcinoma of thoracic esophagus: recurrence pattern and indication for adjuvant therapy

**DOI:** 10.1186/1749-8090-8-S1-O88

**Published:** 2013-09-11

**Authors:** W Fang, X Guo, T Mao, Z Gu

**Affiliations:** 1 Department of Thoracic Surgery, Shanghai Chest Hospital, Jiaotong University Medical School, Shanghai, China

## Background

Management strategy is still undecided for radically resected squamous cell carcinoma of thoracic esophagus free of lymph node metastasis. Analysis of recurrence pattern in this subgroup of patients may help determining necessity and modality of adjuvant therapy.

## Methods

From a prospectively entered database, data of 112 patients proved to be node-negative after esophagectomy with selective three-field lymphadenectomy was retrospectively studied.

## Results

Recurrence happened in 45 patients (40.2%) after operation. Median time to recurrence was 17.4 months. Locoregional relapse was the major recurrence type, detected in 38 patients (33.9%). Distant metastasis alone occurred in only 3 patients (2.7%). All locoregional relapse were in lymph nodes except for 1 (2.6%) at the anastomosis. Altogether, lymph node metastasis accounted for 82.2% of all recurrence. In the 37 cases of lymph node metastasis, the most common sites were neck (56.8%) and superior mediastinum (24.3%). Locoregional relapse was significantly related to tumor location (upper-mid thoracic 41.1% vs. lower thoracic 11.1%), cell differentiation (poor 40% vs. mid-well 25.5%), and depth of invasion (T3-4a 47.1% vs. T1-2 13.6%) (p<0.05). Upon multivariate analysis, tumor location (p=0.049, RR 1.092) and depth of invasion (p=0.017, RR 3.296) were revealed as independent risk factors, with cell differentiation of borderline significance (p=0.068, RR 1.613).

## Conclusions

Although completely resected together with extended lymph node clearance, 40% of the patients free of lymph node involvement at the time of surgery would still develop recurrence. And the most common recurrence pattern was still locoregional, mainly involving lymph nodes in the cervico-thoracic junction. Greater risk of local relapse is associated with higher location of tumor and deeper depth of invasion. Thus, adjuvant therapy should be studied in high risk patients, including those with poor differentiation tumors.

